# Analysis of characteristics of intracranial cavernous angioma and bleeding factors in middle-aged and elderly patients

**DOI:** 10.3389/fneur.2023.1084911

**Published:** 2023-02-06

**Authors:** Chao Xiu, Donghe Ni, Jincui Zhao, Yang Yu

**Affiliations:** ^1^Medical Imaging Center, Affiliated Hospital of Beihua University, Jilin City, China; ^2^Nuclear Magnetic Resonance Department, Integrated Traditional Chinese and Western Medicine Hospital of Jilin Province, Jilin City, China

**Keywords:** middle-aged and elderly, intracranial cavernous angioma, intracranial hemorrhage, related factors, cerebrovascular

## Abstract

**Objective:**

Intracranial cavernous angioma (ICA) is a cerebrovascular malformation. It causes local neurological dysfunction, epilepsy, intracranial hemorrhage (ICH) and other symptoms, seriously affecting the safety of patients. This study analyzed middle-aged and elderly patients with ICA in our hospital, summarized the characteristics of the disease and investigated the related factors of ICH.

**Methods:**

We conducted a retrospective analysis of 120 middle-aged and elderly patients who were diagnosed with ICA by magnetic resonance imaging in our hospital from March 2018 to September 2021. The cases were assigned to either a bleeding group (i.e., the experimental group) or a non-bleeding group (i.e., the control group). The characteristics of the disease, including gender, age, number of lesions, form and symptoms of onset, distribution of lesions, blood supply vessels in the lesion area, size of the lesion and presence of bleeding, were summarized and analyzed. The relationship between these factors and ICH was investigated, and the data were analyzed using SPSS 25.0 software.

**Results:**

There were 56 cases in the experimental group and 64 cases in the control group. A univariate analysis showed that gender, age, body mass index, blood lipids, number of lesions, course of the disease, onset of symptoms and disease characteristics were not associated with ICH in the middle-aged and elderly patients with ICA (*P* > 0.05). The maximum diameter, volume, location and blood supply area of the lesions were related to ICA complicated with ICH (*P* < 0.05). A multivariate unconditional logistic regression analysis revealed that the maximum diameter, volume, location and blood supply area of the lesions were independent risk factors for ICH in the middle-aged and elderly patients with ICA. The odds ratio (OR) of the maximum diameter of the lesion was 4.410, the OR of the lesion volume was 7.316, the OR of the lesion site was 7.470, and the OR of the blood supply area was 1.6588.

**Conclusion:**

Intracranial cavernous angioma lesions in middle-aged and elderly patients occur mainly in the supratentorial area, with a small part located in the infratentorial area. The main form of the disease is chronic recurrence. The occurrence of bleeding is related to the size, location and blood supply of the lesion.

## Introduction

Cavernous malformations of the brain (CCMs) are intracranial vascular malformations that exist as single or mixed vascular lesions (1). Intracranial cavernous angioma (ICA) is a type of CCM that is composed of a cluster of abnormal, transparent capillaries surrounded by hemosiderin precipitation. It is a type of cerebrovascular malformation. It has an incidence of 0.4–0.8% and accounts for 10–25% of intracranial vascular malformations, mainly in elderly patients (2). According to the location of the lesion, the clinical manifestations are different but mainly involve dizziness, headaches, seizures, intracranial hemorrhage (ICH) and focal neurological dysfunction at the corresponding location. The biggest risk is recurrent haemorrhagic transformation and acute concurrent ICH (1, 3).

Pathological examination is the gold standard for the diagnosis of ICA, but its invasiveness and risk make it impractical in clinical practice. Because ICA is a vascular malformation with a small vascular diameter and slow blood flow velocity, it is difficult to detect lesions by digital subtraction angiography and other vascular examination methods, which can easily lead to a missed diagnosis or a misdiagnosis (4). With the continuous development of brain magnetic resonance imaging (MRI), the technique's detection of ICA is also increasing. At present, MRI is the main method for clinically diagnosing ICA. Due to discomfort, most patients with ICA are diagnosed by physical examination or cranial MRI examination. Only a small number of people are diagnosed by their own symptoms, with 8–37% of patients having large bleeding lesions (5). Intracranial cavernous angioma can be regarded as a chronic developing disease. Its early detection and intervention may reduce the risk of subsequent complications.

Middle-aged and elderly adults are most vulnerable to the negative effects of cerebral hemorrhage, and their post-bleeding conditions are difficult, with poor prognoses. However, there are insufficient studies on ICA in middle-aged and elderly people in China, and there is a lack of analysis on the regularity and causes of ICH resulting from ICA. Therefore, this study aimed to analyse the characteristics of ICA in middle-aged and elderly people and investigate the regularity and influencing factors of ICH to provide a reference for the selection of intervention therapy.

## 2. Materials and methods

### 2.1. Research subjects

This study included middle-aged and elderly patients with ICA who were treated in our hospital from March 2018 to September 2021. All patients underwent head MRI and computed tomography (CT) examination and were diagnosed with ICA. According to the inclusion and exclusion criteria, 120 cases were finally included. All cases were assigned to either a bleeding group (i.e., the experimental group) or a non-bleeding group (i.e., the control group), as shown in Table 1. This study was approved by the ethics committee of the Affiliated Hospital of North China University (No. 202238).

**Table 1:** Univariate analysis of ICA complicated with intracranial hemorrhage.

**Index**		**Experience group** **(*n* = 56)**	**Control group** **(*n* = 64)**	**χ^2^/*t***	***P*** **-value**
Gender	Male	27	31	0.343	0.557
	Female	29	33		
Age (years ± SD)		61.4 ± 10.07	62.42 ± 8.70	12.999	0.131
BMI (kg/m^2^ ± SD)		21.57 ± 3.91	21.32 ± 0.34	3.484	0.513
Blood lipid status	Hyperlipidemia	11	6	0.171	0.064
	Low high-density lipoprotein	12	7		
	Normal	33	51		
Number of lesions	Single shot	51	60	0.062	0.431
	Multiple	5	4		
Course of disease (years ± SD)		2.33 ± 0.81	2.84 ± 0.34	0.369	0.713
Maximum diameter of focus (mm ± SD)		14.51 ± 8.99	8.92 ± 1.41	4.656	0.041
Lesion volume (mm^3^ ± SD)		2,301.23 ± 110.21	1,402 ± 8.56	21.847	0.001
Lesion location	Frontal lobe	14	20	3.791	0.041
	Temporal lobe	14	13		
	Basal ganglia	7	15		
	Brainstem	7	8		
	On the screen	37	49		
	Under the curtain	10	6		
	Others	8	9		
Blood supply area	Anterior circulation	17	48	4.513	<0.001
	Posterior circulation	7	9		
	Watershed	32	8		
Onset symptoms	Epilepsy	23	22	0.968	0.735
	Headache	21	23
	Nerve function	8	10
	Others	4	9
Onset characteristics	Acute	17	18	1.047	0.892
	Subacute	10	13		
	Chronic	27	33		

“Low high-density lipoprotein” means the high-density lipoprotein (HDL) ≤ 0.91 mmol.

BMI (Body Mass Index) = body weight (kg)/height^2^ (m).

The inclusion criteria were as follows: (1) patients who were treated in our hospital from March 2018 to September 2021, (2) aged over 45 years (i.e., classed as middle-aged or elderly according to the World Health Organization's definition) and (3) with ICA diagnosed by MRI that met the diagnostic criteria based on the 2017 diagnostic guidelines (5).

The exclusion criteria were as follows: (1) patients with severe cardiopulmonary disease and multiple organ dysfunction, (2) complicated with other congenital neurological diseases and (3) with incomplete imaging and blood biochemical data.

### 2.2. Research methods

The case data of 120 middle-aged and elderly patients with ICA diagnosed by MRI and CT in our hospital were analyzed retrospectively. The characteristics of the disease, including gender, age, number of lesions, onset form and symptoms, lesion distribution, lesion area blood vessels, lesion size and presence of concurrent bleeding, were analyzed and summarized. The relationship between gender, age, number of lesions, distribution of lesions, blood supply vessels in the lesion area, lesion size, blood lipids, body mass index (BMI) and ICH was analyzed.

### 2.3. Imaging examination

#### 2.3.1. Head CT examination

The CT scans were performed using a Siemens DeTintion AS 128-layer CT system. The layer distance and thickness were set to 5 mm, respectively, the current was set to 240 mA, and the voltage was set to 100 kV. During the examination, the physician placed the patient in the supine position, with the scanning range from the top of the skull to the skull base. A 1.5-mg/kg injection of iohexol was administered into the right elbow vein of the patient *via* a high-pressure syringe, and threshold-triggered scanning was performed to obtain images in the arterial, venous and delayed phases.

#### 2.3.2. Head MRI examination

A Philips INGENIA 3.0-T magnetic resonance scanner was used for orthogonal circular scanning in our hospital. The scanning sequences included Tl-weighted images (WI), T2WI, FLAIR, DWI, etc. After routine sequence scanning, enhanced scanning was performed from the top of the skull to the skull base. Two senior neurologists reviewed the patient's head MRI results without knowledge of their medical history or clinical information, and the results were recorded.

### 2.4. Observation indicators

(1) Lesion size: This was determined with reference to the consensus on the guidelines for the management of cerebral cavernous malformations published by the Haemangioma Alliance in 2017 for neurosurgery (5). For spherical or quasi-spherical lesions, the longest diameter was used for description and comparative analysis. To reduce errors, the maximum diameter of each focus was measured in the sagittal, coronal and horizontal planes, and the volume was calculated using the multi-field formula. In cases with multiple lesions and no haemorrhagic disease, the maximum focus was used as statistical data, while in cases with bleeding, the bleeding focus was used.
(2) Onset form: The definition standard was based on the eighth edition of *Diagnostics*, published in 2013 by the People's Health Publishing House: An acute course was within 2 weeks of onset, a subacute course was within 2 weeks to 3 months of onset, and a chronic course was more than 3 months from onset (6).
(3) Onset symptoms: These included seizures, headaches, neurological dysfunction and others.
(4) Lesion location: Based on *Neurology*, the third edition published by the People's Health Publishing House, the location was divided into the frontal lobe, temporal lobe, parietal lobe, occipital lobe, basal ganglia, thalamus, brainstem and cerebellum. The basal ganglia area included the internal capsule, caudate nucleus, lenticular nucleus, tabular nucleus and amygdala. The brain stem included the midbrain, pons and medulla oblongata. For cases with multiple lesions and no haemorrhagic disease, the largest lesions were used as statistical data, while for bleeding cases, bleeding lesions were utilized as statistical data (7).
(5) Focus blood supply area: Based on the third edition of 8-year neurology published in 2015 by the People's Health Publishing House, the focus blood supply area was divided into three areas: anterior circulation, posterior circulation and watershed (8).
(6) Blood lipids: The standard definition of dyslipidaemia was total cholesterol > 5.72 mmol, or triglycerides > 1.70 mmol/L, or low-density lipoprotein > 3.64 mmol or high-density lipoprotein < 0.91 mmol (9).
(7) Body mass index: This was calculated according to the square of weight/height (international unit kg/m^2^). The BMI of the hospitalized patients was divided into three groups according to Chinese reference standards: <18.5, 18.5–23.9, and >23.9 kg/m^2^ (10).
(8) The number of lesions was 1 for a single lesion and ≧2 for multiple lesions.
(9) Intracranial hemorrhage was diagnosed by MRI and CT (11). The MRI imaging findings were as follows: The lesion was irregular in shape and round; the signal was a mixed high and low signal, with a low signal in the surrounding area, including in the haemosiderin ring, with popcorn-like lesions with obvious characteristics. If a small amount, multiple, repeated bleeding, imaging signal performance varied, uneven. The CT imaging findings were as follows: The lesion was irregular in shape and round. The density was either high or slightly high, with equal density shadow or mainly uneven density. Generally, there was no oedema around, no mass effect and no lower mass effect. When the lesion was bleeding, the lesion increased rapidly, and the occupying effect appeared. With enhanced scanning, there was no enhancement or significant to mild enhancement.

All cases were assigned to either the bleeding group (i.e., the experimental group) or the non-bleeding group (i.e., the control group), and the differences between the two groups were observed and analyzed. The relationship between gender, age, number of lesions, distribution of lesions, blood supply vessels in the lesion area, lesion size, blood lipids, BMI and concurrent ICH was analyzed statistically.

### 2.5. Statistical methods

We used SPSS 25.0 software for the statistical analysis of the data. Quantitative data were analyzed by the Q–Q plot formula for normal distribution, data consistent with a normal distribution were described by mean ± standard deviation, and a *t*-test was used for comparisons between groups. An inconsistent normal distribution was expressed by the median value, and a rank–sum test was used for comparisons between groups. Qualitative data were described as relative numbers and were compared between groups using the Chi-squared test. Data with a sample size of ≤40 or >20% of cells with a theoretical frequency of <5 or 0 per cell were analyzed using Fisher's exact test. Using ICH as the dependent variable and the statistically significant factors in the univariate analysis as independent variables, stepwise regression was included in the binary logistic regression equation to analyse the influencing factors of ICH in middle-aged and elderly patients with ICA. The test level was α = 0.05.

## 3. Results

### 3.1. Imaging analysis of the patients with ICA

A total of 120 patients with ICA were examined for a single focus in 111 cases and multiple foci in nine cases. The distribution was mainly in the supratentorial, frontal, and temporal lobes. A cavernous haemangioma was a lobulated vascular mass or a red circle, similar to a mulberry or a strawberry.

#### 3.1.1. The CT findings

Among the 120 patients, the tumor density was uneven in 30 cases; there was calcification in 34 cases and bleeding in 56 cases. Among the patients with bleeding, the edge of the focus in 19 patients was a circular or approximately circular structure, while the edge of the focus in 12 patients was irregular. The lesion boundary was unclear in 15 patients, while it was clear in 10 patients.

#### 3.1.2. The MRI findings

**Figure 1:**
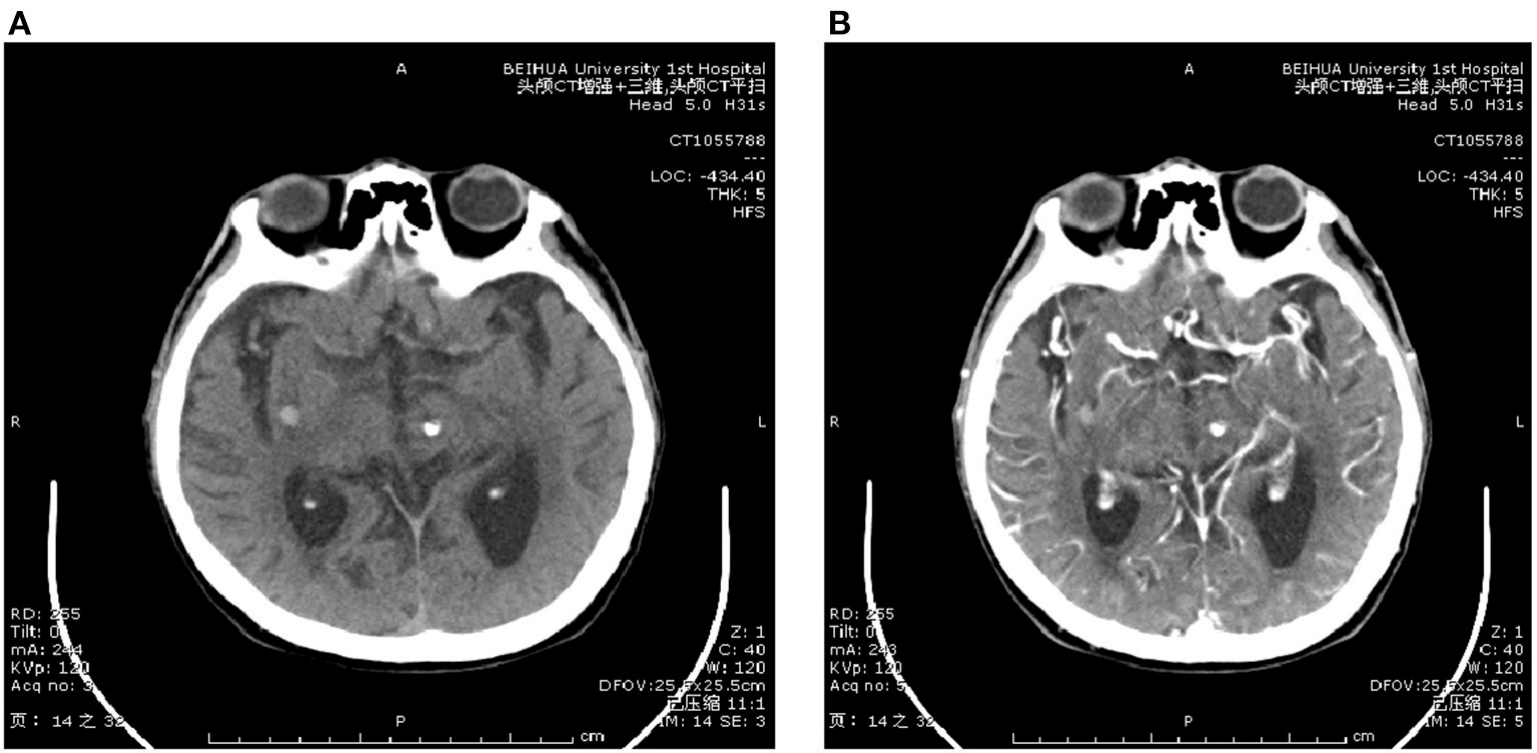
CT scanning image. **(A)** Shows CT plain scan image and **(B)** shows CT enhanced image.

**Figure 2:**
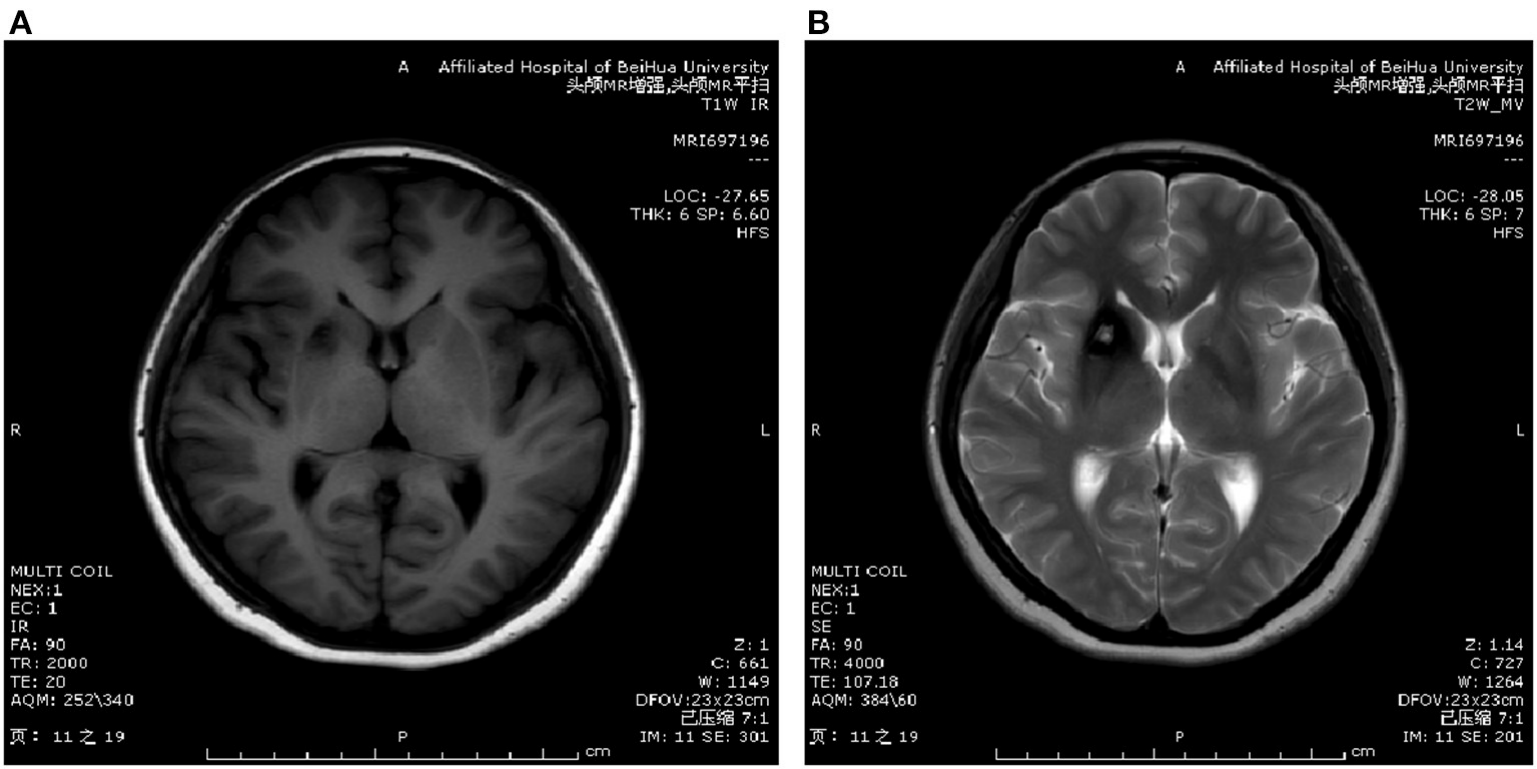
MRI scanning image. **(A)** Shows T1WI image and **(B)** shows T2WI image.

On the plain MRI scans, 25 cases of T1WI mixed signals, 12 cases of high signals, 76 cases of low signals and seven cases of equal signals were observed. The T2WI results showed high signals in 19 cases, mixed signals in 27 cases and low signals in 74 cases. Thirty-two patients had a typical semi-ring signal and a T2WI ring signal, and 12 patients had cornflower changes (see Figures 1, 2).

### 3.2. Univariate analysis of ICA complicated with ICH

A total of 120 middle-aged and elderly patients with ICA were included and analyzed. They were assigned to either the experimental group or the control group according to whether they were bleeding or not. There were 56 cases of bleeding and 64 cases of non-bleeding. There were 27 (48.21%) males and 29 (51.79%) females in the experimental group, with an average age of 61.4 ± 10.07 years and a BMI of 21.57 ± 3.91 kg/m^2^. There were 31 (48.44%) males and 33 (51.56%) females in the control group, with an average age of 62.42 ± 8.70 years and a BMI of 21.32 ± 0.34 kg/m^2^.

The univariate analysis results showed that gender, age, BMI, blood lipids, number of lesions, course of the disease, onset symptoms and characteristics were not associated with ICH in the middle-aged and elderly patients with ICA (*P* > 0.05). The maximum diameter and volume of the lesions in the experimental group (14.51 ± 8.99 mm) were significantly larger than those in the control group (8.92 ± 1.41 mm) (*P* < 0.05). The lesions occurred mostly on the curtain (37 vs. 49), frontal lobe (14 vs. 20) and temporal lobe (14 vs. 13), and there was a significant difference between the two groups (*P* < 0.05). The blood supply area of the lesions in the experimental group was mainly the watershed area (57.14%), while in the control group, it was mainly anterior circulation (75.00%). The difference between the two groups was statistically significant (*P* < 0.05) (see Table 1). The maximum diameter, volume, location and blood supply area of the lesion were related to ICA complicated with ICH (*P* < 0.05) (see Table 1).

### 3.3. Multivariate logistic regression analysis of ICA complicated with ICH

The results of the multivariate unconditional logistic regression analysis showed that the maximum diameter, volume, location and blood supply area of the lesion were independent risk factors for ICH in the middle-aged and elderly patients with ICA, and the difference was statistically significant (odds ratio = 4.41, 7.32, 7.47, and 1.66, respectively, *P* < 0.05), as shown in Table 2.

**Table 2:** Multivariate logistic regression analysis of ICA complicated with intracranial hemorrhage.

**Influence factor**	**B value**	**SE value**	**Wald value**	**OR (95%CI) value**	***P-*** **value**
Maximum diameter of focus	2.24	0.53	10.91	4.41 (3.34–19.69)	0.007
Lesion volume	1.67	0.50	11.37	7.32 (2.01–14.04)	0.001
Lesion location	2.01	0.51	15.68	7.47 (2.76–20.21)	0.001
Blood supply area	0.70	0.06	26.86	1.66 (1.37–2.01)	<0.001

## 4. Discussion

Intracranial cavernous angioma is also known as cavernous tumor or haemangioma. Its prevalence is about 0.16–0.5% (12). Common clinical manifestations include seizures, ICH and focal neurological deficit without imaging evidence of recent bleeding, with probabilities of 50, 25, and 25%, respectively. Some patients have no obvious abnormal symptoms. According to the results of different statistical studies, asymptomatic patients account for 20–50% of the total number of cases (13). The lack of typical clinical features makes clinical diagnosis difficult.

Characteristic of ICA, plain CT scans reveal a clear boundary of circular or round slightly high-density shadow, previously attributed in the literature to calcification (14–16). In this study, 19 patients had a round or nearly round structure at the edge of the lesion. Research suggests that oedema around a tumor has a certain value for determining the presence of fresh bleeding in the tumor. Hu et al. (17) believe that there is no mass effect in ICA. In this study, although the size and shape of the tumors were different, there was no mass effect; therefore, the no-mass effect was an important feature in the CT signs of ICA. After a contrast-enhanced scan, there was a certain difference between intracerebral and extracerebral cavernous haemangioma. The former had no obvious enhancement or only mild uneven enhancement, while the enhancement in the latter was obvious, which was consistent with the results of this study.

The pathology of cerebral hemorrhage has been compared in young and middle-aged mice. The results showed that the neurological function of middle-aged mice was reduced along with the activation of astrocytes (18). Therefore, the present study selected elderly patients as research subjects, i.e., those over 45 years of age. A total of 120 cases of ICA were collected, with a total of 56 cases of bleeding. The proportion of males and females was close to 1:1, and no gender tendency was found. The main clinical manifestations were recurrent seizures, headache, dizziness and focal neurological dysfunction. There were more supratentorial lesions than infratentorial lesions. Most of the lesions were located in the area of anterior circulation and the watershed. The statistical analysis revealed the complication of ICH was related not to the patient's gender, age, number of lesions, blood lipids and BMI but to the size, location and blood supply of the lesion. The reasons for this are as follows.

The results showed that the bleeding rate of supratentorial ICA was higher than that of other parts. This is consistent with the results recorded in the literature (19). As an abnormal vascular disease, the pathology of ICA is a type of abnormal capillary mass. Although many studies have confirmed that lesions in the brain stem are prone to bleeding, it is difficult to explain this propensity simply from the localization of the brain lobe. This study suggests that it may be more reasonable to locate according to blood vessels; therefore, the second localization method can be performed based on lobar localization, i.e., according to the location and classification of the blood supply arteries at the location of the focus, the intracranial vessels can be subdivided and summarized into the anterior circulation blood supply area, the posterior circulation blood supply area and the watershed area (20). The results revealed that the bleeding probability of lesions in the anterior circulation and posterior circulation areas was low, while the bleeding probability of lesions in the watershed area was high. After the statistical analysis, the difference between each group was statistically significant; it is considered that when the lesions are distributed in the watershed area, the probability of ICA complicated with ICH is higher than that in other parts.

Essentially, the watershed area is a junction area dominated by multiple blood vessels that contain capillary networks to communicate with different blood vessels (21). This area is prone to a lack of blood supply due to various factors, resulting in ischemia and necrosis of vascular tissue. After the blood supply is restored, it will stimulate the formation of new capillaries. Therefore, ICA located in the watershed area often degenerates, necrotises and regenerates repeatedly, resulting in the enlargement of the tumor and the mixing of new and necrotic capillaries, thereby destabilizing the structure of the focus so that it is easily complicated with bleeding (22). Second, ICA in the watershed area may accept the blood supply of multiple vessels, the pressure and hemodynamics of which are different, especially in the watershed area between the anterior and posterior circulation (23). This makes the internal hemodynamics of ICA in the Shuiling area significantly different from those in other parts, resulting in vascular wall degeneration, increased permeability and bleeding. Idiculla et al. (1) revealed that the coexistence of ICA and developmental venous anomalies increases the risk of bleeding.

There have been few studies on lesion size in ICA. Most of the cases collected in the present study were spherical or quasi-spherical, and the longest diameter was used as the comparison standard. The maximum diameter and volume of the lesions in the experimental group and the control group were compared and analyzed. The statistical analysis revealed that the difference between the groups was statistically significant. Combined with the analysis of the results, considering the correlation between the size of the lesion and the incidence of ICA complicated with ICH in young people, the longest diameter and volume of a lesion may be a reference point for predicting whether ICH is complicated, as reported by Adamski et al. (24). When a lesion is large, the larger the blood flow it accommodates and the more disordered the pressure in the lesion, which may lead to the degeneration and rupture of the vascular wall and bleeding when the acute pressure changes. In addition, the lesion size in ICA may be a gradual process throughout the entire course of the disease. With repeated micro bleeding, small vessel injury and neocapillaries, the size of the ICA may increase, and the old and new capillaries will be mixed, which, inevitably, will lead to the decline of the structural stability of the lesion and increase the possibility of bleeding transformation in the case of emergency. Schuss et al. (25) demonstrated a propensity for epilepsy in patients with ICA with lesions located in the temporal lobe.

The choice of treatment and the identification of residual disease during surgery are extremely important. The use of postoperative adjunctive techniques facilitates identification, particularly intracranial angiography. In clinical practice, it may be due to the location of the malformed vascular mass located on the brainstem in some patients, resulting in inoperability due to the difficulty and high risk of surgery. For such patients, the regular review of head CTA is required to identify lesions, and intracranial angiography is required when necessary. For patients treated conservatively, intracranial angiography needs to be perfected during hospitalization to identify the size of the malformed vascular mass, the condition of the draining veins, and whether it is associated with aneurysms. Then, after combining these various factors, appropriate treatment can be selected.

### 4.1. Limitations

Due to the short timescale of this study, only 120 patients in our hospital were included, the sample size was small, and there was selection bias. Other research has shown that ICA has asymptomatic bleeding, although we did not analyse this factor in our study. In the future, we will conduct a multi-center large-sample study to investigate other vascular abnormalities to enable us to draw clear conclusions.

## 5. Conclusion

In this study, the incidence of cavernous haemangioma in middle-aged and elderly patients was mainly sporadic and single. The clinical manifestations were mostly chronic recurrent seizures, headaches and dizziness. Lesions were distributed mainly in the supratentorial area and watershed blood supply area. The maximum diameter of the lesion, its volume, location and blood supply area were independent risk factors for middle-aged patients with ICA complicated with ICH. In this paper, the clinical characteristics of ICA in 120 middle-aged and elderly patients were summarized and described to provide a basis for subsequent research and treatment.

## Data availability statement

The original contributions presented in the study are included in the article/supplementary material, further inquiries can be directed to the corresponding author.

## Ethics statement

The studies involving human participants were reviewed and approved by the Ethics Committee of Affiliated Hospital of North China University. The patients/participants provided their written informed consent to participate in this study.

## Author contributions

CX and YY conceived the study and helped to draft the manuscript. CX participated in its design, data analysis, and statistics. DHN and JCZ helped revised and proofread the manuscript. All authors read and approved the final manuscript.
